# Kinetic and thermodynamic characterization of the reaction pathway of box H/ACA RNA-guided pseudouridine formation

**DOI:** 10.1093/nar/gks882

**Published:** 2012-09-24

**Authors:** Xinxing Yang, Jingqi Duan, Shuang Li, Peng Wang, Shoucai Ma, Keqiong Ye, Xin Sheng Zhao

**Affiliations:** ^1^Beijing National Laboratory for Molecular Sciences, State Key Laboratory for Structural Chemistry of Unstable and Stable Species, Department of Chemical Biology, College of Chemistry and Molecular Engineering, and Biodynamic Optical Imaging Center, Peking University, Beijing 100871 and ^2^National Institute of Biological Sciences, Beijing 102206, China

## Abstract

The box H/ACA RNA-guided pseudouridine synthase is a complicated ribonucleoprotein enzyme that recruits substrate via both the guide RNA and the catalytic subunit Cbf5. Structural studies have revealed multiple conformations of the enzyme, but a quantitative description of the reaction pathway is still lacking. Using fluorescence correlation spectroscopy, we here measured the equilibrium dissociation constants and kinetic association and dissociation rates of substrate and product complexes mimicking various reaction intermediate states. These data support a sequential model for substrate loading and product release regulated by the thumb loop of Cbf5. The uridine substrate is first bound primarily through interaction with the guide RNA and then loaded into the active site while progressively interacted with the thumb. After modification, the subtle chemical structure change from uridine to pseudouridine at the target site triggers the release of the thumb, resulting in an intermediate complex with the product bound mainly by the guide RNA. By dissecting the role of Gar1 in individual steps of substrate turnover, we show that Gar1 plays a major role in catalysis and also accelerates product release about 2-fold. Our biophysical results integrate with previous structural knowledge into a coherent reaction pathway of H/ACA RNA-guided pseudouridylation.

## INTRODUCTION

Pseudouridine (Ψ), the most abundant modified nucleotide present in rRNAs, tRNAs and snRNAs, is converted posttranscriptionally from uridine (U) at specific sites of RNA by pseudouridine synthases (ΨSs) (1,2). The modification replaces a N1-glycosidic bond in U with a C5-glycosidic bond in Ψ and results in only a subtle change in the chemical structure of the base, an extra hydrogen donor N1. According to sequence homology, ΨSs are classified into six families, named after the representative members, TruA, TruB, RluA, RsuA, TruD and Pus10. Structural studies have shown that the catalytic domains of ΨSs share a familiar fold and active site configuration, suggesting that Ψ formation is governed by a common catalytic mechanism. All ΨSs contain an invariant, catalytically essential aspartate at the active site (Asp85 in *Pyrococcus furiosus* Cbf5), but the exact catalysis mechanism remains unclear (3–6).

Most ΨSs are composed of single polypeptides and specify substrate RNAs by protein recognition. In contrast, the H/ACA-RNA guided ΨS is a complicated ribonucleoprotein particle (RNP) composed of a distinct H/ACA guide RNA and four proteins Cbf5, Nop10, L7Ae and Gar1 (7–10). Enzymatically active H/ACA RNPs have been reconstituted with recombinant proteins and transcribed RNAs in both archaeal and eukaryotic systems (11–13). Cbf5 is the catalytic subunit closely related to the tRNA Ψ55 synthase TruB (14–17). The basic unit of H/ACA RNA folds into a hairpin structure with a large internal loop. The loop can form two about 6-bp duplexes with substrate sequences flanking the U to be modified (18). The H/ACA RNA associates with L7Ae, Nop10 and Cbf5 such that the substrate-binding guides are placed at one side of the active site cleft of Cbf5 (19), whereas Gar1 binds to the catalytic domain of Cbf5 at the other side of the active cleft (16). Although eukaryotic H/ACA RNAs universally possess two hairpin units, each hairpin constitutes the basic structural and functional unit *in vitro* (13).

In the absence of other factors such as helicases, H/ACA RNP is able to turnover substrate by itself (13,20). The enzyme must possess an autonomous mechanism to load substrate and release modified product. H/ACA RNP relies on the guide RNA as well as the catalytic subunit Cbf5 to bind substrates (20,21). Such a substrate-binding mode implicates that H/ACA RNP possesses a more complicated mechanism of substrate recruitment and product release compared with stand-alone ΨSs.

Structural analyses of H/ACA RNP and its substrate/product complexes have revealed multiple conformations of the enzyme. These studies highlight the thumb loop of Cbf5 as a key mobile element involved in substrate recruitment. In the absence of substrate, the thumb adopts an open conformation with its N-terminal root region docked at Gar1 and its tip region disordered (19). The H/ACA RNP structure obtained with a substrate containing 5-fluorouridine as modification target is regarded to mostly mimic the reactive state (20,21). In this structure, the 5-fluorouridine has been converted into the hydrolyzed product 5-fluoro-6′-hydroxyl pseudouridine (5FhΨ), as observed in the TruB structure (14), and the thumb interacts extensively with the product-like RNA in a fully ordered, closed conformation (20,21). More recently, Zhou *et al.* determined several H/ACA RNP structures bound with substrates containing nonreactive U analogues 5′-bromouridine and 3′-methyluridine or modified products of reactive analogues 4′-thiouridine and 2′-deoxyuridine (22,23). The structures with nonreactive targets may represent some prereactive states, whereas the structures with modified targets may resemble postreactive states. These structures revealed intermediate conformations of the thumb with its root adopting a closed-like conformation and its tip region disordered. The nonreactive target nucleotide is partially loaded at the active site, yet the modified nucleotide of reactive analogues is disordered.

Previous structural studies have provided snapshots of H/ACA RNP in different functional states. However, a full understanding of the substrate turnover mechanism of H/ACA RNP requires quantitative knowledge about the stability of each reaction intermediate and the kinetic rates of individual reaction step. Moreover, all substrate-bound H/ACA RNP structures determined so far were obtained with U analogues at the target site, it is unclear to what extent these structures reflect the binding mode of normal U substrates and Ψ products.

In this study, we characterized the interaction of H/ACA RNP with U substrate and Ψ product RNAs by measuring the apparent equilibrium dissociation constants (*K*_d_) and kinetic dissociation (*k*_off_) and association (*k*_on_) rates using fluorescence correlation spectroscopy (FCS). We took advantage of mutant Cbf5 proteins to block the reaction at specific steps. Our results reveal a sequential model of substrate loading and product release in H/ACA RNA-guided pseudouridylation. We further analyzed the role of Gar1 in individual steps of substrate turnover.

## MATERIALS AND METHODS

### Expression, purification and assembly of H/ACA RNP

*Pyrococcus furiosus* (Pf) H/ACA RNPs were assembled from one molar equivalence of Cbf5-Nop10 subcomplex, Gar1 and the RNA1 H/ACA RNA and two molar equivalences of L7Ae in 50 mM phosphate buffer (pH 7.6) and 1 M NaCl, as previously described (19,20). The DEL7 mutant of Cbf5, in which residues 143–152 in the thumb loop were replaced by Gly–Pro–Gly, and the R154Q mutant have been described previously (20). The D85A mutation was introduced using the QuikChange method. The ΔGar1 complexes were assembled without Gar1.

### Substrate RNA analogues

Substrate RNAs with 3′-end labeled DY547 were purchased from Dharmacon, deprotected following manufacturer’s instructions and dissolved in water. The substrate Sub-U has the sequence 5′-AUAAUU*U*GACUCAA-3′, where the target U is in italic. The substrates Sub-Ψ and Sub-C have the same sequence as Sub-U except that the target U at position 7 is replaced by Ψ and cytosine, respectively.

In this study, Sub-Ψ was prepared by enzymatic modification of Sub-U. Specifically, DY547-labeled Sub-U (20 µM) was incubated with 1 µM H/ACA RNP in 50 µl of reaction buffer containing 50 mM phosphate (pH 7.6) and 1 M NaCl at 37°C for 12 h. The modification should be 99.999% complete after 12 h according to the reaction rate previously measured at the same condition (20). The reaction mixture was separated in a 15% denaturing urea polyacrylamide gel. The gel band containing fluorescent RNA was excised and crushed. The RNA was soaked out in 200 µl reaction buffer at room temperature for 1 h. The soaking solution was centrifuged and passed through a 0.22 µm filter to remove small gel particles.

### Enzymatic activity

The pseudouridylation activity of H/ACA RNP was measured as described previously (20). The solutions of single-turnover reactions containing 6 μM WT-RNP or ΔGar1-RNP, ∼0.1 nM singly ^32^P-labeled substrate and 2 μM unlabeled substrate were incubated at 27°C or 37°C. The multiple-turnover reactions were conducted with 2 μM ΔGar1-RNP and 2, 4 or 8 μM substrates at 37°C. The reaction mixture was digested by RNase A/S7 mixture into mononucleotides, which were separated with thin layer chromatography and visualized with autoradiography.

### Fluorescence correlation spectroscopy

The FCS experiments were conducted on a modified Nikon TE2000 microscopy as previously described (24). The samples were excited by a CW 532 nm laser (SUW Tech., China) with 300 μW of power. The fluorescence signal was collected with an oil-immersion 100 × objective (1.4 N.A. Nikon, Japan), divided with a nonpolarizing 50/50 splitter (XF121, Omegafilter, USA) and recorded using two avalanche photo diodes (SPCM-AQR-14, Perkin-Elmer, USA). The autocorrelation function was recorded in a manner of cross correlation with a computer-implemented correlator (Flex02-12D, www.correlator.com). The fluorescence signal was collected for 30, 60 or 180 s depending on signal-to-noise ratio. All FCS data were collected at 27°C. The temperature was maintained by a home-built temperature controller. All reactions were carried out in binding buffer of 50 mM phosphate (pH 7.6), 1 M NaCl and 0.02% Tween-20. All thermodynamic and kinetic measurements were repeated at least twice.

### FCS data analysis

In our binding reactions, the fluorescence labeled substrate is in either free or RNP-bound states. The FCS function *G*(t) is determined by a two-component diffusion function
(1)
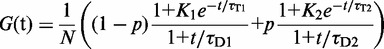

where *p* is the percentage of bound substrate, *N* is the average total number of dye-labeled substrate molecules in the monitored confocal volume, *τ*_D1_ and *τ*_D2_ are the characteristic diffusion times of free and RNP-bound substrates, *K*_1_ and *τ*_T1_ are the equilibrium constant and relaxation time describing the photophysical process of DY547 in free substrate and *K*_2_ and *τ*_T2_ are the corresponding parameters in RNP-bound substrate. For every combination of substrate and enzyme, the parameters *K*_1_, *τ*_T1_, *K*_2_, *τ*_T2_, *τ*_D1_ and *τ*_D2_ were first determined from control experiments with 100% free substrate (*p* = 0) or ∼100% bound substrate (*p* = 1) in the presence of 5–10 μM RNP. These parameters were fixed in subsequent fitting processes, whereas *N* and *p* were adjustable in fitting. The nonlinear fitting of FCS curves were conducted in Matlab 2010 a (MathWorks).

### Kinetic association rates

Equal volumes of H/ACA RNPs (0.1–1 μM final concentration) and DY547-labeled substrates (10 nM final concentration) were mixed and the fluorescence signal was monitored for 10–30 min. The FCS curve and the fraction of bound substrate were derived as described above. The fraction of bound substrate *p* was fitted to a single-exponential function 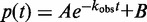 to derive the apparent rate of binding reaction *k*_obs_. The association experiments were conducted with 3–4 concentrations of enzyme (0.1–1μM). The averaged *k*_obs_ values from two independent measurements at each protein concentration were fit to the linear equation
(2)
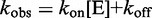

where [E] is the concentration of H/ACA RNP, *k*_on_ is the association rate and *k*_off_ is the dissociation rate. Since *k*_off_ would be poorly determined from the intercept, it was determined by kinetic dissociation experiments as described below.

### Kinetic dissociation rates

To measure the dissociation rates, substrate RNAs (2 μM) were incubated with H/ACA RNPs (6 μM) at 27°C for 60 min for nonreactive combinations. The mixture was rapidly diluted with binding buffer to a final substrate concentration of 0.5 nM, at which complexes would almost completely dissociate into substrate RNA and H/ACA RNP. The dissociation of substrate was continuously monitored by FCS every 30, 60 or 180 s until a plateau was reached. The curves were fitted by single-exponential decay function, 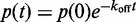, where *k*_off_ is the dissociation rate and *p*(0) is the fraction of bound RNA at *t* = 0. To clearly compare the dissociation rates of different samples, the normalized fraction of bound RNA, *p*(*t*)/*p*(0), was plotted in Figure 3.

### Kinetic dissociation analysis of slowly reacting substrate–enzyme complexes

Sub-U (2 μM) was incubated with ΔGar1-RNP (6 μM) from 5 to 293 min prior to dilution. Upon 4000-fold dilution, dissociation of unmodified substrate, modification reaction and dissociation of product entangled together, as shown in the theme below.
(3)
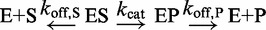

where S is the substrate, P is the modified product, E is the enzyme, *k*_cat_ is the modification rate, and *k*_off, __S_ and *k*_off, __P_ are the dissociation rate of substrate and product, respectively. ES represents the most stable substrate complex prior to dissociation, which corresponds to ES2 shown in Figure 7A. EP refers to the most stable product complex prior to dissociation, corresponding to EP1 in Figure 7A.

In the case of Sub-U being catalyzed by ΔGar1-RNP, the modification reaction was much slower than the formation of ES complex. After initial mixing of Sub-U with ΔGar1-RNP, ES was quickly formed, and the catalytic reaction proceeded subsequently. As the enzyme concentration used was much greater than the equilibrium dissociation constant of the reactant complex, *K*_d,__S_, the substrate should be essentially all enzyme-bound before dilution, namely [S] = 0. After an incubation time of *T*, the total concentration of unmodified substrate was 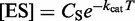 and the total concentration of product was 
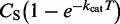
, where *C*_S_ is the initial concentration of the substrate. The product was partitioned in the free and RNP-bound states with 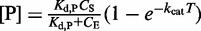 and 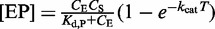, where *C*_E_ is the total concentration of H/ACA RNP and *K*_d,__P_ is the equilibrium dissociation constant of product complex. Since the final concentrations of substrate and product after dilution were very low (0.5 nM), the re-association could be ignored.

Based on reaction (3), we derived a formula to account for the dissociation of substrate, dissociation of product, and ongoing modification
(4)
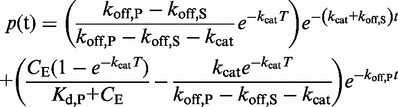

where *p*(t) is the total fraction of bound substrate and product RNAs, *T* is the incubation time prior to dilution, and *t* is the time after dilution.

The total fraction of bound S and P was measured by FCS as described before. All dissociation curves collected with different *T* were globally fit to a double-exponential function: 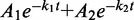. The decay rates *k*_1_ and *k*_2_ were kept the same for all dissociation curves and the amplitudes *A*_1_ and *A*_2_ were variable for curves of different incubation times. According to Equation (4), the fast decay rate *k*_1_ corresponds to *k*_off,__P_, and the slow decay rate *k*_2_ is the sum of *k*_cat_ and *k*_off,__S_. *k*_cat_ can be determined by fitting the amplitudes of pre-exponential components *A*_1_ or *A*_2_ to a single-exponential function of *T.* The mean and standard deviation of *k*_cat_ values obtained from *A*_1_ and *A*_2_ fitting were reported.

### Equilibrium dissociation constants

DY547-labeled substrate RNAs (10 nM) were incubated with H/ACA RNP from 10 nM to 10 µM for 30 min at 27°C before FCS analysis. To derive the apparent dissociation constant *K*_d_, the fraction of bound substrate (*p*) was fitted to equation
(5)
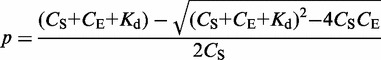

where *C*_S_ and *C*_E_ are the total concentration of substrate and H/ACA RNP, respectively. The mean and standard deviation of *K*_d_ values from 2–3 independent measurements were reported.

## RESULTS AND DISCUSSION

### Kinetic and thermodynamic parameters of H/ACA substrate–enzyme complexes

To study the reaction pathway of H/ACA RNA-guided Ψ synthesis, we employed FCS to monitor the binding of substrate or product RNA to the well characterized Pf H/ACA RNP in real time or at equilibrium conditions. The cognate substrate RNA with a U at the target site (Sub-U) was chemically synthesized with a fluorescent dye DY547 at the 3′-end and the corresponding product RNA (Sub-Ψ) was prepared by enzymatic modification of Sub-U. These RNAs were assembled with wild-type (WT) enzyme or its variants to mimic intermediates along the reaction path.

DY547-labeled Sub-Ψ was also chemically synthesized. However, for unknown reason, the chemically synthesized Sub-Ψ binds H/ACA RNP with lower efficiency compared with the enzymatically prepared Sub-Ψ. Since the enzymatically prepared Sub-Ψ is the real product of H/ACA RNP, its binding parameters are most relevant. The results with the chemically synthesized Sub-Ψ were not considered.

**Figure 1.**
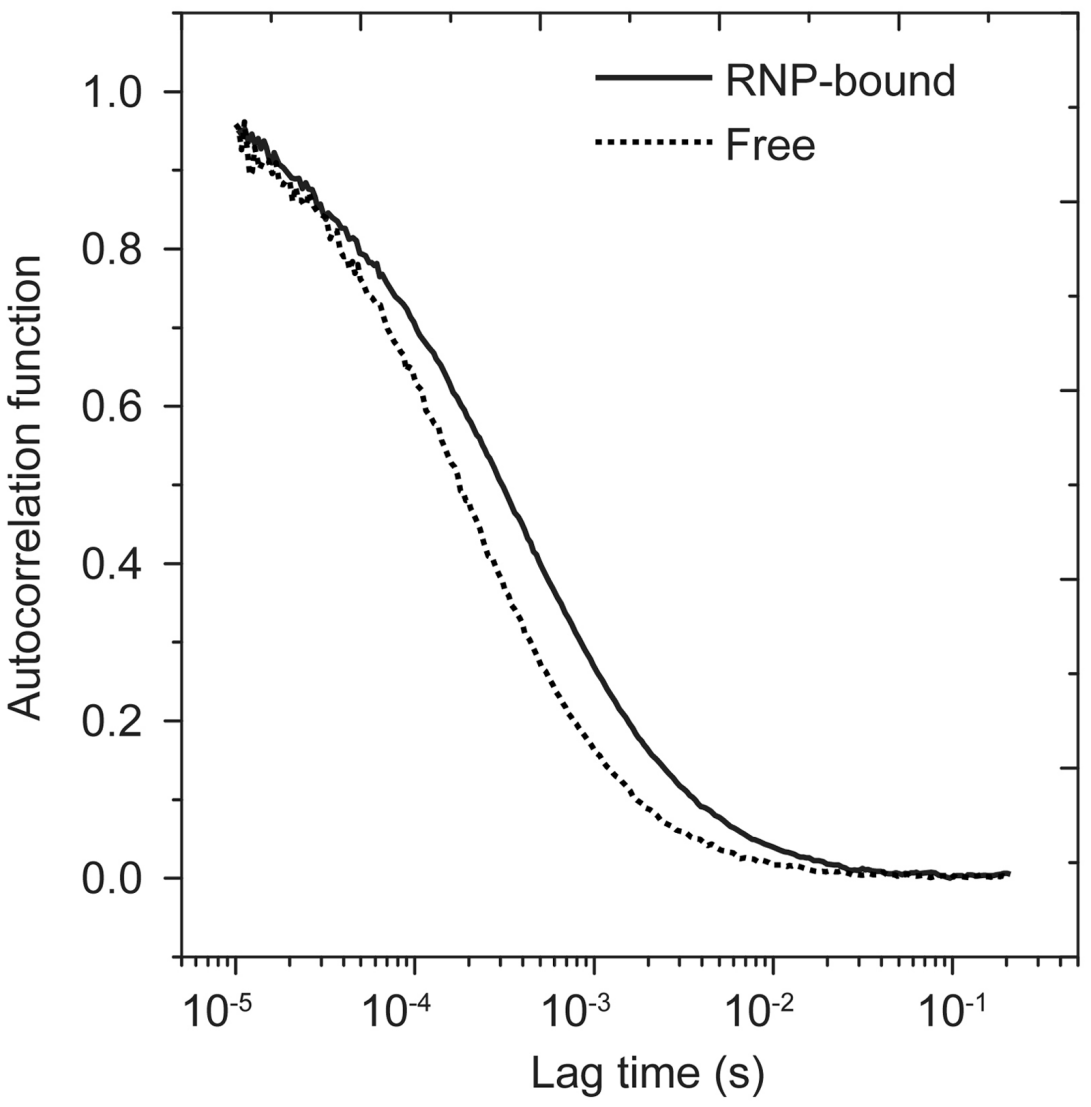
The fluorescence autocorrelation functions of uridine substrate alone and that bound to D85A-RNP. The curves were fitted to Equation (1) (*p* = 0 or *p* = 1), yielding a diffusion time *τ*_D_ of 0.21 ms for free substrate and 0.47 ms for RNP-bound substrate.

In FCS, the fluorescence fluctuations of substrate RNA were recorded from a tiny volume (∼1 fl) of sample over 1–3 min. The diffusion constant of substrate RNA can be derived from the autocorrelation function (FCS curve) of fluorescence fluctuations. The free and RNP-bound substrates, which differ greatly in molecular weight (5 kD versus 100 kD), show well separated diffusion times *τ*_D_ (0.21 versus 0.47 ms) (Figure 1). After the diffusion and photophysics parameters of free and bound substrate were determined, the fraction of bound substrate in a mixed sample can be obtained by fitting FCS curves to Equation (1).

**Figure 2.**
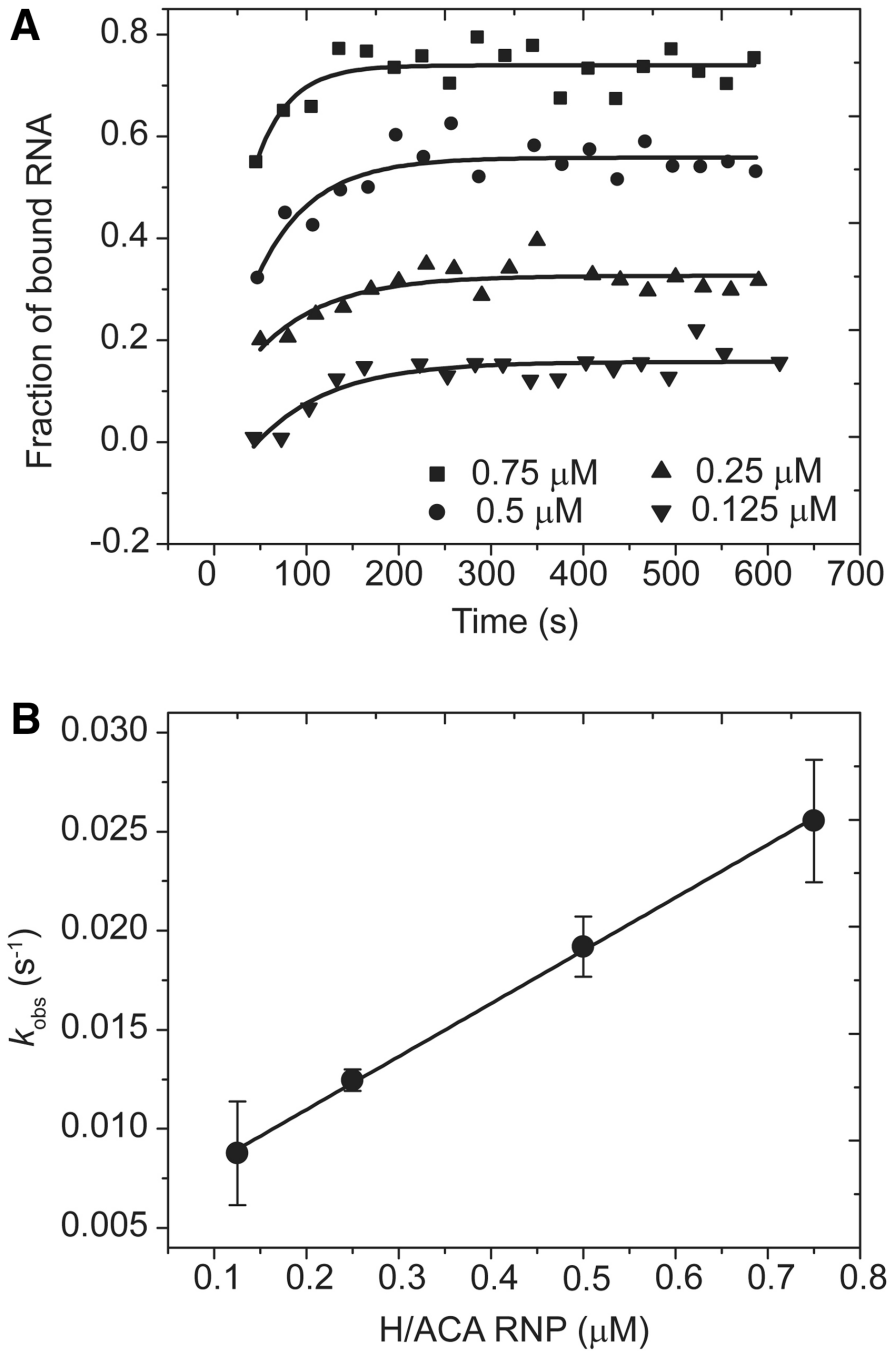
Representative measurements of kinetic association rate. (**A**) The time course of association of Sub-U and WT-RNP. Sub-U (10 nM) was assembled with WT-RNP of 0.125, 0.25, 0.5 or 0.75 μM. FCS was measured over time and the fraction of bound RNA was derived. The curves are the best single-exponential fit, yielding apparent binding rates *k*_obs_. (**B**) The mean values and SD of *k*_obs_ from two measurements are plotted as a function of H/ACA RNP concentration. The line is the best linear fit with the association rate (slope) *k*_on_ = 0.0267 ± 0.0005 μM^−1^ s^−1^.

**Figure 3.**
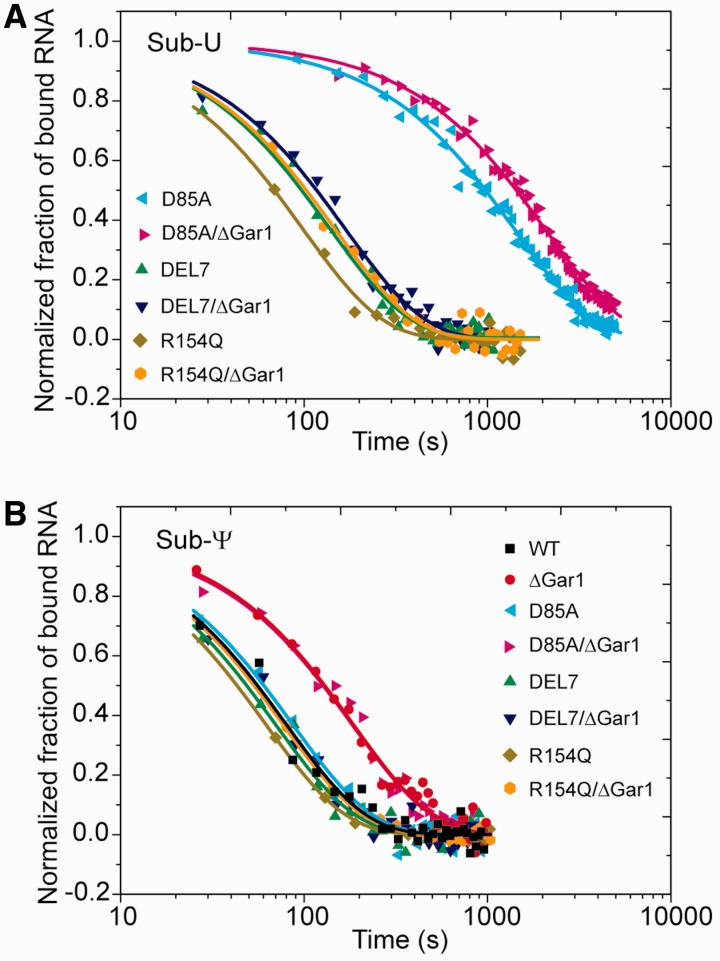
Dissociation of H/ACA substrate–enzyme complexes. Sub-U (**A**) and Sub-Ψ (**B**), each at a 2 μM concentration, were incubated with WT-, D85A-, DEL7- and R154Q-RNP and their ΔGar1 counterparts (6 μM) for 60 min at 27°C before 4000-fold dilution. Each dissociation data set was fitted to a single-exponential function and the normalized fraction of bound RNA is displayed.

**Figure 4.**
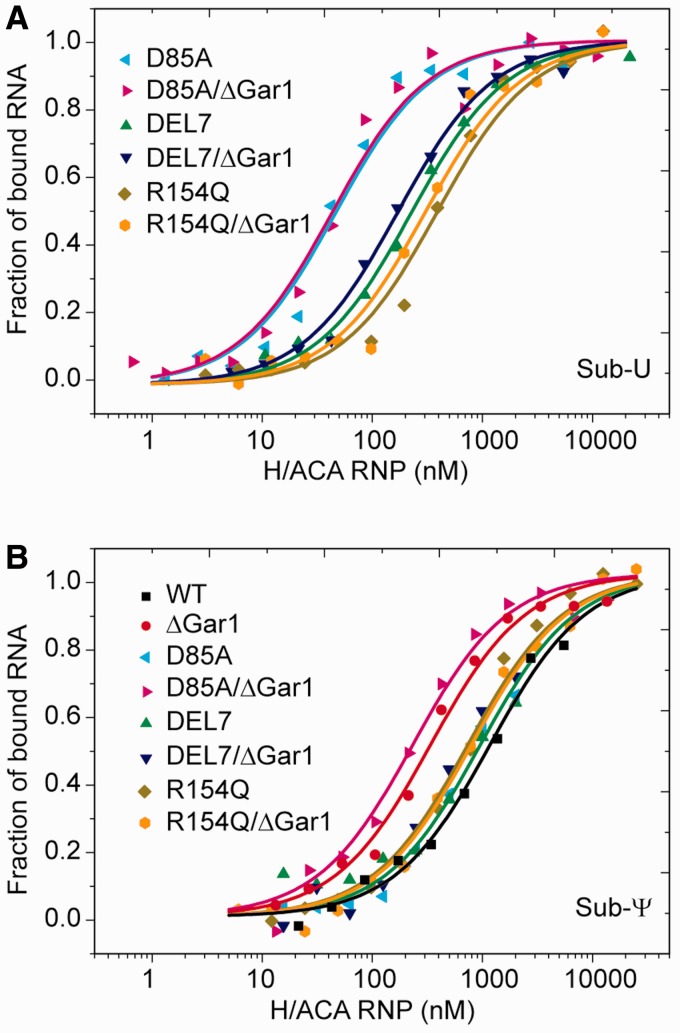
Equilibrium dissociation constants measured by titrating substrate RNA with H/ACA RNPs. Sub-U (**A**) and Sub-Ψ (**B**), each at a 10 nM concentration, were incubated with WT-, D85A-, DEL7- and R154Q-RNP and their ΔGar1 counterparts of increasing concentrations for 30 min at 27°C. The fractions of bound substrates obtained from FCS analysis were fit to Equation (5) to derive the apparent dissociation constant *K*_d_.

The kinetics of substrate binding to H/ACA RNP was examined by following the FCS signal of substrate in real time after addition of H/ACA RNP (Figure 2A). The binding rate *k*_obs_ obtained by a single-exponential fit increased linearly with the concentration of H/ACA RNP (Figure 2B). According to Equation (2), the slope of this increase represents the association rate *k*_on_ of substrate (Table 1). To measure the dissociation rate *k*_off_, the preassembled substrate–enzyme complex was rapidly diluted and the fraction of bound substrate was measured by FCS in real time. The dissociation curve was normally analyzed by a single-exponential fit (Figure 3, Table 1). In addition, the thermodynamic stability of various substrate complexes was measured by titrating substrate RNA with H/ACA RNP of increasing concentrations. The fraction of bound RNA was fitted to Equation (5) to obtain the apparent equilibrium dissociation constant *K*_d_ (Figure 4, Table1). As FCS is sensitive to molecular size, the equilibrium and kinetic data were analyzed with a simple two-state binding model (free and bound substrates) and the apparent parameters were derived.

**Table 1.** The apparent equilibrium and kinetic binding parameters between H/ACA RNPs and substrate RNAs at 27°C

Substrate	H/ACA RNP	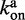 (10^3 ^M^−1^ s^−1^)	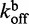 (10^−3^ s^−1^)	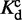 (µM)
Sub-U^d^	WT	27 ± 1	n.d.	n.d.
Sub-U	D85A	17 ± 1	0.69 ± 0.09	0.031 ± 0.002
Sub-U	DEL7	18 ± 3	12 ± 3	0.23 ± 0.08
Sub-U	R154Q	11 ± 3	11 ± 2	0.47 ± 0.05
Sub-U	RNA only	n.d.	27 ± 5	1.5 ± 0.1
Sub-Ψ	WT	23 ± 5	12 ± 1	1.1 ± 0.2
Sub-Ψ	D85A	22 ± 4	11 ± 1	0.9 ± 0.1
Sub-Ψ	DEL7	15 ± 3	14 ± 1	0.9 ± 0.1
Sub-Ψ	R154Q	18 ± 8	16 ± 2	0.8 ± 0.1
Sub-Ψ	RNA only	n.d.	17 ± 5	1.72 ± 0.15
Sub-U^d^	ΔGar1	12 ± 1	n.d.	n.d.
Sub-U	ΔGar1/D85A	26 ± 1	0.54 ± 0.04	0.036 ± 0.002
Sub-U	ΔGar1/DEL7	8.4 ± 0.4	7.8 ± 0.7	0.15 ± 0.01
Sub-U	ΔGar1/R154Q	8 ± 2	7 ± 1	0.42 ± 0.06
Sub-Ψ	ΔGar1	27 ± 5	5 ± 1	0.32 ± 0.04
Sub-Ψ	ΔGar1/D85A	35 ± 4	5 ± 1	0.20 ± 0.03
Sub-Ψ	ΔGar1/DEL7	22 ± 3	12 ± 1	0.7 ± 0.1
Sub-Ψ	ΔGar1/R154Q	24 ± 11	16 ± 4	0.83 ± 0.09
Sub-C	WT	n.d.	42 ± 9	3.5 ± 0.1

^a^The errors of *k*_on_ are from fitting.

^b^*k*_off_ values are the mean ± SD from 2–3 independent measurements.

^c^*K*_d_ values are the mean ± SD from 2–3 independent measurements.

^d^These substrate–enzyme combinations are reactive. Due to the fact that the concentrations of substrate and product RNAs vary with time, the apparent *K*_d_ and *k*_off_ would be time dependent and, therefore, cannot be determined by conventional analysis, n. d., not determined.

### Dissociation of reactive H/ACA substrate–enzyme complexes

**Figure 5.**
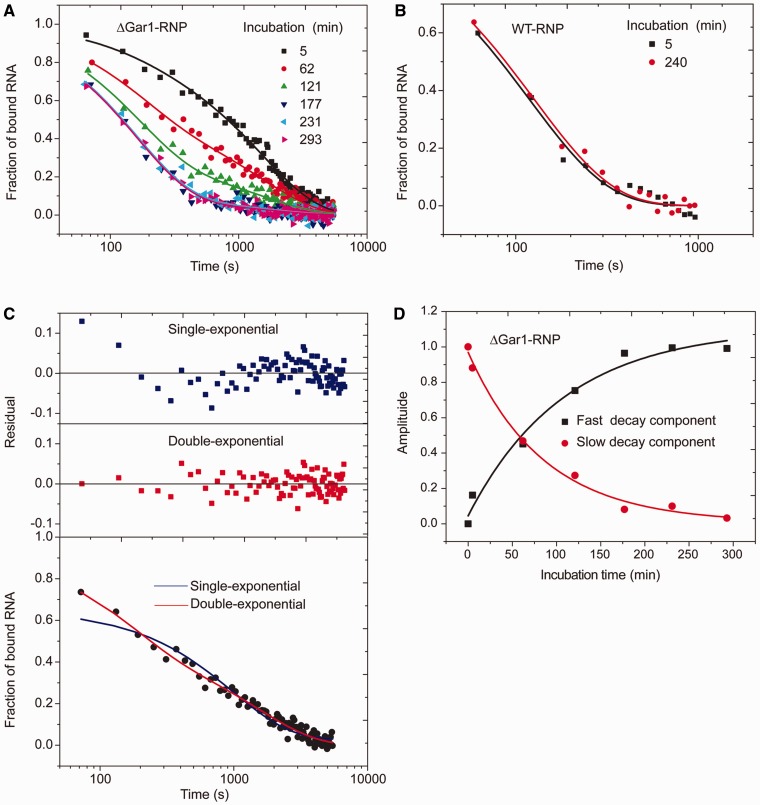
Dissociation of substrate from reactive H/ACA RNPs. (**A**) Dissociation curves of the Sub-U/ΔGar1-RNP complex. After incubation of 5, 62, 121, 177, 231 and 293 min at 27°C, the mixture of Sub-U (2 µM) and ΔGar1-RNP (6 µM) was rapidly diluted by 4000-fold. The fractions of bound substrate and product RNAs are plotted against the dissociation time. The curves are the best global fit to a double-exponential function. (**B**) Dissociation curves of the Sub-U/WT-RNP complex. The reaction was incubated for 5 min or 4 h prior to dilution. Other experimental settings were the same as those in (A). (**C**) Comparison of single-exponential (blue line) versus double-exponential (red line) fitting of a dissociation curve of Sub-U with ΔGar1-RNP (*T* = 62 min). The fitting residuals of two models are shown on the top. (**D**) The amplitudes of fast (black square) and slow (red circle) decay components for dissociation of Sub-U/ΔGar1-RNP complex are plotted as a function of incubation time *T*. The lines are the best fit to a single-exponential decay.

The WT-RNP and ΔGar1-RNP enzymes are active toward the U substrate, which would complicate the measurement and interpretation of binding parameters. We found that the dissociation rate of Sub-U from ΔGar1-RNP was dependent on incubation time before onset of dilution. When incubation time was extended, the dissociation speed was accelerated until reaching a plateau (Figure 5A). This phenomenon suggests that modification by ΔGar1-RNP was not complete at onset of dissociation and longer incubation led to more products that likely dissociate faster than unmodified substrates.

In contrast, the dissociation kinetics of Sub-U from WT-RNP was identical whether the incubation time was 5 min or 4 h (Figure 5B), suggesting that majority of the substrate had been modified by WT-RNP shortly after mixing. The measured dissociation rates of Sub-U and Sub-Ψ from WT-RNP were indeed the same (Tables 1 and 2).

**Table 2.** Kinetic parameters of reactive substrate and H/ACA RNPs at 27°C

Substrate	H/ACA RNP	*k*_off.S _(10^−3^ s^−1^)	*k*_off.P _(10^−3^ s^−1^)	*k*_cat _(10^−3^ s^−1^)	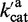 (10^−3^ s^−1^)
Sub-U	WT	n.d.	12 ± 1	n.d.	3.9 ± 0.6
Sub-U	ΔGar1	0.45 ± 0.04	6.4 ± 0.5	0.19 ± 0.05	0.08 ± 0.02

^a^Estimated by single-turnover activity assay, n.d., not determined.

**Figure 6.**
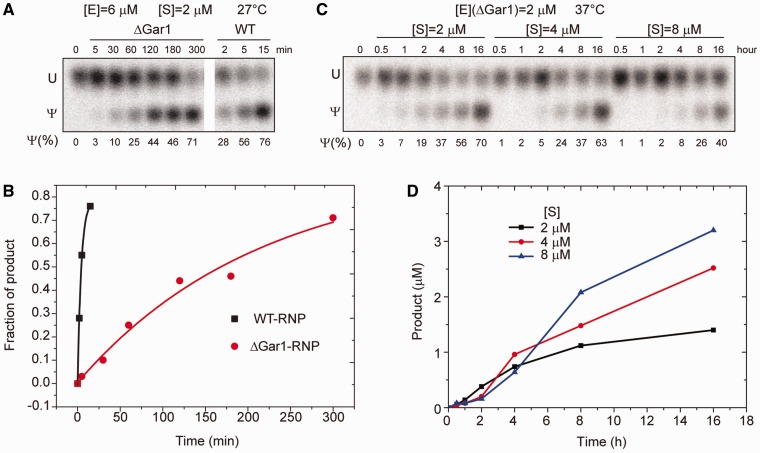
Pseudouridylation activity of H/ACA RNPs. (**A**) Single-turnover activity of WT- and ΔGar1-RNP at 27°C. The Sub-U (2 µM) was labeled with a single ^32^P at the 3′-end of target U and incubated with WT- and ΔGar1-RNP H/ACA RNP (6 µM) for indicated times. The product was digested into 3′-P mononucleotides that were then separated by thin layer chromatography and visualized by autoradiography. (**B**) The fraction of product is plotted against reaction time for reactions in (A). The lines are the best fit to a single-exponential function with 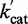 of 7.8 × 10^−5^ s^−1^ for ΔGar1-RNP and 3.9 × 10^−3^ s^−1^ for WT-RNP. (**C**) Multiple turnover reactions of ΔGar1-RNP. ΔGar1 H/ACA RNP (2 μM) was incubated with 2, 4 or 8 μM substrates at 37°C. (**D**) The concentration of product is plotted against the reaction time for reactions in (C).

To assess the degree of modification at onset of dissociation, we measured the activity of WT- and ΔGar1-RNP in the same conditions as those used during incubation period of dissociation experiments (Figure 6A and B). In these single-turnover reaction conditions, WT-RNP modified half of substrate in 2.8 min, whereas the defective ΔGar1-RNP modified half of substrate in 148 min. These results confirm the above interpretation of dissociation data of reactive H/ACA RNPs.

The low reactivity of ΔGar1-RNP indicates that the dissociation experiment monitors three concurrent events: dissociation of unmodified substrate, dissociation of modified product and modification. In this case, FCS measured the total fraction of bound substrate and product RNAs. According to Equation (4), the dissociation of slow reacting complexes can be described by two exponential components: a slow decay component that accounts for dissociation of unmodified substrate as well as modification and a fast decay component that accounts for dissociation of modified product.

Indeed, the dissociation data of the Sub-U/ΔGar1-RNP complex cannot be properly fitted by a single-exponential function, but they were satisfactorily fitted by a double-exponential function (an example is given in Figure 5C). As the incubation time was increased, the amplitude of fast decay component increased and the amplitude of slow decay component concurrently decreased, indicating an increased amount of product generated over the time (Figure 5D). By double-exponential global fitting of the dissociation curves (Figure 5A) and single-exponential fitting of the amplitudes of fast and slow decay components (Figure 5D), we obtained dissociation rates of 4.5 × 10^−^^4^ s^−^^1^ for unmodified substrate and 6.4 × 10^−^^3^ s^−^^1^ for modified product and an apparent reaction rate *k*_cat_ of 1.9 × 10^−^^4^ s^−^^1^ for ΔGar1-RNP (Table 2). The dissociation rate of Sub-U from reactive enzyme is otherwise difficult to measure. The reaction rate of ΔGar1-RNP measured from dissociation experiments is slightly faster than that measured from single-turnover activity experiments. The systematic and random deviations of different detection approaches may contribute to the observed differences in the measured reaction rates. We think the value from the dissociation experiment is more accurate, because its measurement is experimentally more direct and instantaneous without separation and purification.

### The U substrate is bound significantly tighter than the Ψ product

As a multiple-turnover enzyme, H/ACA RNP should be able to attract unmodified substrate and discharge modified product. We asked whether U substrates are bound with higher affinity than Ψ products. The product–enzyme complex can be generated by assembling Sub-Ψ and WT-RNP. The Sub-U and WT-RNP complex cannot be studied by our FCS method because WT-RNP quickly converts Sub-U to Sub-Ψ, whereas the poorly active Sub-U and ΔGar1-RNP complex can be studied as shown above. To mimic the substrate–enzyme complex, the catalytic residue Asp85 of Cbf5 was mutated to alanine (12,25). The resultant D85A-RNP is inactive in catalysis but appears to be a good approximation of WT-RNP in terms of substrate and product binding, as suggested by available data. First, D85A- and WT-RNP exhibit nearly identical *k*_on_, *k*_off_ and *K*_d_ values for Ψ product RNAs (Figures 3 and 4, Table 1). Second, the *k*_off_ of Sub-U with ΔGar1-RNP (0.45 × 10^−^^3^ s^−^^1^) measured from the dissociation experiments (Figure 5 and Table 2) is also comparable with that of ΔGar1/D85A-RNP (0.54 × 10^−^^3^ s^−^^1^).

The equilibrium *K*_d_ data show that Sub-Ψ (*K*_d_ = 0.9 µM) is bound to D85A-RNP 29-fold weaker than Sub-U (*K*_d_ = 0.031 µM) (Figure 4, Table 1). The reduced binding affinity of Sub-Ψ to D85A-RNP is mainly ascribed to its increased dissociation rate (16-fold). Moreover, dissociation analysis of slowly reacting ΔGar1-RNP demonstrates that the modified product is released from the active enzyme 14-fold faster than the unmodified substrate (Table 2). These data indicate that H/ACA RNP can discriminate the subtle change of chemical structure at the target site and strongly favor U against Ψ.

### Interaction of the thumb contributes to different binding affinities of U substrate and Ψ product

The large difference of binding affinity between U substrate and Ψ product suggests that they are recognized in different mode at their most stably bound states. We asked what the structural basis for this difference is. The thumb loop of Cbf5 has been shown to be an important substrate-binding element (19–23). To assess the contribution of the thumb in binding substrate and product RNA, we analyzed H/ACA RNP assembled with the DEL7 or R154Q mutant of Cbf5 (Figures 3 and 4). The DEL7 mutant lacks the tip region of the thumb that is critical for pinching the substrate RNA at the active cleft. The R154 residue is located at the C-terminal root part of the thumb and interacts with a phosphate group of substrate. Both mutants have been shown to completely abolish the activity and they likely block the substrate from adopting the catalysis competent conformation (20).

Compared with D85A RNP, the two mutant RNPs show large increase of *K*_d_ (7- to 14-fold) and *k*_off_ (17-fold) for Sub-U. These results indicate that the thumb is required for stable association of Sub-U at the most stable state. Most likely, the thumb adopts a closed conformation in the U substrate complex, as observed in the 5FhΨ product structure (20,21).

In contrast, Sub-Ψ has nearly identical *K*_d_ and *k*_off_ values for H/ACA RNPs assembled with WT, D85A, DEL7 and R154Q Cbf5 (Figures 3 and 4, Table 1). This suggests that the thumb has virtually no energetic contribution to the binding of Ψ product in the context of fully assembled enzyme at the most stable state. We can conclude that the lack of thumb interaction accounts for the weak binding of Ψ product.

### A kinetic intermediate state during substrate loading

Our thermodynamic and mutagenesis data have shown that the thumb is required for high affinity binding of U substrate at equilibrium condition. In contrast, in the kinetic association experiments that were based on FCS detection and monitored the formation of substrate encounter complex, we found that Sub-U bound WT-, D85A-, R154Q- and DEL7-RNP with similar association rates (Table 1). These results suggest that when the substrate is initially associated, a kinetic intermediate is formed that is largely devoid of thumb interaction. In addition, Sub-Ψ was found to bind with similar rates as Sub-U, suggesting that the target base is not discriminated in the initial bound state.

### A sequential model for substrate binding and product release

**Figure 7.**
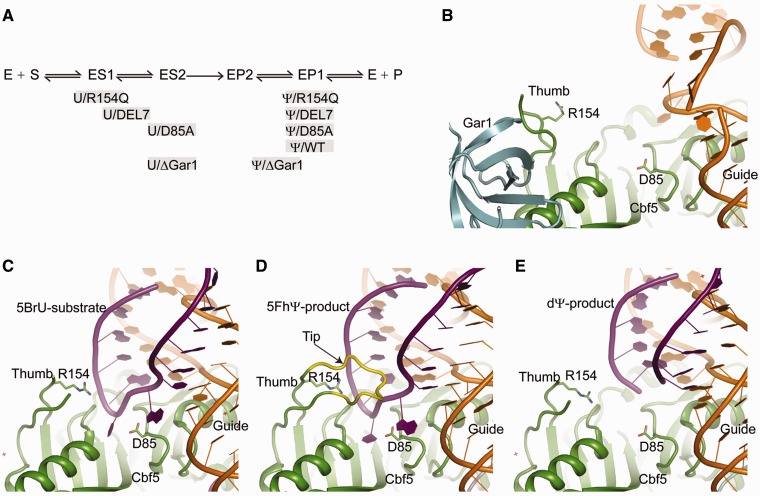
Substrate turnover model in H/ACA RNA-guided pseudouridylation. (**A**) A simplified two-step model for substrate loading and product release. The U substrate (S) first assembles with the enzyme (E) into an intermediate ES1, which then converts into the reactive complex ES2 upon folding of the thumb. After modification, the product (P) is initially bound in the reactive-like EP2 complex, which transforms into the EP1 complex after release of the thumb. Various substrate/product complexes formed with different RNP mutants are placed along the reaction pathway at positions approximate to their mimic reaction intermediates. (**B**) Structure of the fully assembled substrate-free H/ACA RNP (PDB code 2HVY). (**C**) Structure of ΔGar1 H/ACA RNP in complex with a 5-bromouridine (5BrU)-containing substrate (PDB code 3LWO). (**D**) Structure of ΔGar1 H/ACA RNP in complex with a 5FhΨ-containing substrate (PDB code 2HAX). The thumb tip deleted in DEL7 mutant is shown in yellow. (**E**) Structure of ΔGar1 H/ACA RNP in complex with a 2′-deoxypseudouridine (dΨ)-containing substrate (PDB code 3LWV).

Our kinetic and thermodynamic data of H/ACA substrate/product complexes support a sequential model for substrate loading as well as product release, as illustrated in Figure 7A. The substrate complex ES2 and product complex EP2 are two analogous states present immediately before and after modification. The model also depicts an intermediate substrate complex ES1 leading to ES2 and an intermediate product complex EP1 converted from EP2.

The reactive state ES2 can be mimicked by the U substrate bound to D85A-RNP at equilibrium condition. Although no crystal structure is currently available for H/ACA RNP bound with a U substrate, the tight binding of substrate and the dependence of binding affinity on the thumb suggest that ES2 resembles the 5FhΨ complex with a characteristic closed conformation of the thumb (Figure 7D). The formation of ES2 complex appears to have a strict requirement for the chemical structure of base at the target site. The nonreactive 5-bromouridine and 3-methyluridine substrates have been shown not to assemble into such a reactive state (22,23). The substrate with a cytosine at the target site apparently cannot form a tight ES2 complex either, as evidenced by its poor binding affinity (Table 1).

Our kinetic data point that the substrate first assembles into an intermediate ES1 that is distinguished from the reactive ES2 state by a lack of dependence on the thumb. The substrate appears to be initially recruited primarily through base pairing interaction with the guide RNA. It has been shown that the association between guide and substrate RNAs can occur in the absence of protein contact (26–28).

The transient ES1 state is expected to transform into the reactive ES2 state via additional intermediates as the substrate establishes more interactions with Cbf5. Initial substrate–protein contacts likely occur at the root part of the thumb and the active site. The substrate becomes fully loaded after the tip of the thumb is closed. The substrate complex of DEL7-RNP (*K*_d_ = 0.23 µM) may be considered an intermediate prior to the closing of the thumb tip, whereas the less stable substrate complex of R154Q-RNP (*K*_d_ = 0.47 µM) may represent an early ES1-like intermediate in which the interaction with the thumb root has not yet formed. The guide RNA appears to mediate majority of interaction with substrate in R154Q-RNP, since the substrate RNA binds to the isolated guide RNA with an only moderately increased *K*_d_ (1.51 µM) and *k*_off_ (0.027 s^−^^1^) compared with R154Q-RNP (Table 1). Crystal structures of H/ACA RNP bound with nonreactive substrates containing 5-bromouridine or 3-methyluridine have provided insight into intermediate states with partially loaded substrates (22,23). In these structures, the substrate interacts with the active site cleft and the root part of thumb, but not with the tip region of the thumb (Figure 7C).

The catalysis rate constants for three different type ΨSs TruB, RluA and TruA were recently determined to be all around 0.5 s^−^^1^ at 37°C (29). This rate is two orders of magnitude faster than the H/ACA RNP single-turnover reaction rate (*k*_cat_') of 0.004 s^−^^1^ measured at 27°C (Figure 6A). Several factors may account for this difference. First, the reaction temperature is 10 degree lower in our assay. Second, Cbf5 catalyzes intrinsically slower than TruB, but this seems less likely given that the two proteins are closely related in sequence and structure. Third, the single-turnover rate we measured is also determined by substrate loading, which may be the rate limiting step.

After modification, the product is bound, at least transiently, in the reactive-like EP2 state with a closed thumb. Structural studies have shown that the modified 5FhΨ product can be stably trapped in the reactive-like state (Figure 7D). However, for normal Ψ products, EP2 appears to be unstable and quickly transforms into EP1, a marginally stable intermediate represented by the Ψ product complex. The thumb should be largely released from product RNA in EP1, since the DEL7 and R154Q mutations, which disrupt the thumb interaction with substrate, have no effect on the stability of the Sub-Ψ/WT-RNP complex. Moreover, the Ψ product RNA binds to the isolated guide RNA with *K*_d_ of 1.72 µM and *k*_off_ of 0.017 s^−^^1^, similar to those of the Sub-Ψ/WT-RNP complex (Table 1). This further supports that the binding of Ψ product RNA in EP1 is largely mediated by the guide RNA. The H/ACA RNP structures bound with modified products of 4-thiouridine or 2-deoxyuridine have provided a reasonable model for EP1 state (23). In these structures, the modified target is discharged from the active site cleft and the thumb is largely released from the product RNA (Figure 7E).

The Pf H/ACA RNP is from a hyperthermophile and most active around 60–70°C (11,12), while our experiments were conducted at 27°C. Although the binding parameters would be dependent on temperature, the derived reaction pathway that is determined by the nature of substrate and product interaction with H/ACA RNP should be conserved at all temperatures.

Our model is based on *in vitro* analysis of one given H/ACA RNP and one given short substrate. It is conceivable that the substrate turnover process would be more complex in cell. The association and dissociation of large rRNA substrates are likely additionally affected by the degree of complementarity between substrate and guide, the folding tendency of substrate, and helicase action.

### Role of Gar1 in individual steps of substrate turnover

Early genetic studies in budding yeast have shown that Gar1 is required for rRNA pseudouridylation (30). Biochemical studies using reconstituted archaeal H/ACA RNPs further indicated that Gar1 is important for single- and multiple-turnover activities (11,12,20). Gar1 binds the catalytic domain of Cbf5 near the root of the thumb loop. Based on the structural observation that part of the thumb loop is docked at Gar1 when the substrate is not present (Figure 7B), it was previously proposed that Gar1 facilitates product release by pulling the thumb off from the product (19). However, it was difficult to dissect the contribution of Gar1 to individual substrate turnover steps, namely substrate loading, catalysis, and product release, based on previous mutagenesis and activity measurement (20). To directly assess the contribution of Gar1 in individual steps of substrate turnover, we examined how removal of Gar1 affects binding of substrate and product RNAs (Table 1).

In single-turnover reactions that do not require product release (Figure 6A), removal of Gar1 dramatically (53-fold) inhibited the reaction rate, consistent with previous observations (11,12,20). Clearly, Gar1 is crucial for the substrate loading and/or catalysis step. To assess the specific contribution of Gar1 to substrate loading, we examined the binding of Sub-U to H/ACA RNPs assembled without Gar1. We found that removal of Gar1 in D85A-RNP caused little change on the *K*_d_, *k*_on_ and *k*_off_ values of Sub-U, suggesting that the substrate loading step is minimally affected by the absence of Gar1. Moreover, our FCS experiments directly showed that deletion of Gar1 substantially reduced the rate of catalytic reaction (Figure 5). We can conclude that Gar1 deletion primarily inhibits the catalysis step. As Gar1 does not constitute the active site or directly contact with the substrate, its effect on catalysis should be indirect. The interaction of Gar1 with the N-terminal root part of the thumb (around Ile139 and Ile140) is persistent in the substrate-loaded state (20) and may be important for the thumb to maintain an optimal configuration of substrate and active site that reduces the activation barrier of reaction.

We further assessed the contribution of Gar1 to product release by analyzing the binding of Sub-Ψ to H/ACA RNPs assembled without Gar1. The deletion of Gar1 from WT- or D85A-RNP consistently reduced the dissociation rate of Sub-Ψ by 2.2- or 2.4-fold and increased the binding affinity of Sub-Ψ by 3.4- or 4.5-fold. These results provide the most direct evidence so far that Gar1 plays a role in accelerating product release.

In mechanistic terms, Gar1 binds to the N-terminal root of the thumb (residues 139–145) in the absence of substrate (19). This interaction may counteract the interaction between the thumb and product RNA, facilitating product release. In the absence of Gar1, the product RNA probably makes residual interaction with the thumb and is stabilized in an intermediate state that proceeds EP1 (Figure 7A). Moreover, the dissociation and stability of the DEL7- or R154Q-RNP product complex were not affected by the absence of Gar1, further supporting that the action of Gar1 in product release is mediated by the thumb. The thumb has been previously shown to be needed for Gar1 to affect substrate positioning (31).

Our data also show that Gar1-mediated acceleration of product release is moderate (2- to 4-fold) and not as great as previously thought. According to this scenario, ΔGar1-RNP should retain some substrate turnover activity. Indeed, we observed a very weak substrate turnover activity for ΔGar1-RNP after 8 h of incubation at 37°C (Figure 6C and D). To partially overcome extremely low activity of ΔGar1-RNP, these reactions were carried out at 37°C since the single-turnover rate is 8-fold faster at this temperature than at 27°C (data not shown).

The action of Gar1 appears to be specific for Ψ product, since the binding affinity and dissociation rate of U substrate are insignificantly affected by Gar1 deletion. The U substrate is bound tightly by the closed thumb in ES2 state at equilibrium condition. The interaction of Gar1 with the thumb may be not strong enough to counteract the strong interaction of U substrate with the thumb.

## CONCLUSION

We have dissected the reaction pathway of H/ACA RNP-mediated Ψ formation by characterizing the kinetic and thermodynamic properties of reaction intermediate mimics. These results, corroborated with previous structural studies of reaction intermediates, lead to a sequential model of substrate loading and product release. The substrate is first loaded primarily through contact with the guide RNA and then transforms into the reactive state upon forming progressive interactions with Cbf5, particularly with the thumb loop. Conversely, after modification the Ψ product first triggers the release of the thumb, resulting in an intermediate with marginal stability. We show that Gar1 promotes catalysis by lowering the reaction barrier and facilitates product release by destabilizing the product complex. The stepwise interaction with the thumb plays a key role in controlling substrate turnover in H/ACA RNA-guided pseudouridylation.

## FUNDING

National Key Basic Research Science Foundation [2010CB912302, 2012CB917304 to X.S.Z and 2010CB835402 to K.Y.]; Natural Science Foundation of China [21233002 and 20973015 to X.S.Z.]; Beijing Municipal Government (to K.Y.). Funding for open access charge: Ministry of Science and Technology of China.

*Conflict of interest statement*. None declared.
